# Brooding Is Related to Neural Alterations during Autobiographical Memory Retrieval in Aging

**DOI:** 10.3389/fnagi.2016.00219

**Published:** 2016-09-16

**Authors:** Sophia Schneider, Stefanie Brassen

**Affiliations:** Department of Systems Neuroscience, University Medical Center Hamburg-EppendorfHamburg, Germany

**Keywords:** rumination, late-life depression, subgenual anterior cingulate cortex, emotional memory, self-referential thinking

## Abstract

Brooding rumination is considered a central aspect of depression in midlife. As older people tend to review their past, rumination tendency might be particularly crucial in late life since it might hinder older adults to adequately evaluate previous events. We scanned 22 non-depressed older adults with varying degrees of brooding tendency with functional magnetic resonance imaging (MRI) while they performed the construction and elaboration of autobiographical memories. Behavioral findings demonstrate that brooders reported lower mood states, needed more time for memory construction and rated their memories as less detailed and less positive. On the neural level, brooding tendency was related to increased amygdala activation during the search for specific memories and reduced engagement of cortical networks during elaboration. Moreover, coupling patterns of the subgenual cingulate cortex with the hippocampus (HC) and the amygdala predicted details and less positive valence of memories in brooders. Our findings support the hypothesis that ruminative thinking interferes with the search for specific memories while facilitating the uncontrolled retrieval of negatively biased self-schemes. The observed neurobehavioral dysfunctions might put older people with brooding tendency at high risk for becoming depressed when reviewing their past. Training of autobiographical memory ability might therefore be a promising approach to increase resilience against depression in late-life.

## Introduction

Rumination, especially brooding rumination, defined as the tendency to focus on negative aspects of one’s self or negative interpretations of one’s life, is a well-known risk and key factor of depression in midlife (Whitmer and Gotlib, 2013). It predicts the onset of new episodes (Nolen-Hoeksema, 2000; Abela and Hankin, 2011) and the severity and duration of existing depressive episodes (Nolen-Hoeksema, 1991). Rumination is suggested to consist of the two components brooding and reflection, whereas brooding is thought to be the more maladaptive response style (Treynor et al., 2003; Armey et al., 2009; Gibb et al., 2012). Neurobiologically, brooding rumination has been related to dysfunctions in fronto-limbic circuits, mostly reflected by an increased, “hyperresponsive” amygdala activation (Siegle et al., 2002; Cooney et al., 2010; Mandell et al., 2014) while dorsolateral prefrontal brain regions were frequently found to be decreased in activity in response to emotional stimuli (Ray et al., 2005; Brassen et al., 2012). Both findings have been interpreted in terms of an impaired cognitive control of heightened emotional reactivity (Ray et al., 2005; Woody et al., 2015). Moreover, the consistently increased engagement of the subgenual anterior cingulate cortex (sgACC) during rest in ruminators (Bratman et al., 2015; Hamilton et al., 2015) as well as during rumination induction in depressives (Cooney et al., 2010) has been related to the maladaptive pattern of aggravated self-referential processes typically observable in ruminators (Hamilton et al., 2015).

Autobiographical memory and self-referential processing are intrinsically related (Conway and Pleydell-Pearce, 2000), and negatively biased self-schemes are thought to hinder autobiographical memory retrieval in depression (Dalgleish and Werner-Seidler, 2014) and at-risk persons in younger age (Sperduti et al., 2013; Young et al., 2016). Reduced autobiographical memory ability and specificity have been related to trait rumination in the current (Schoofs et al., 2013; Hamlat et al., 2015) and previously depressed young patients (Raes et al., 2012). Rumination induction in healthy young participants during the presentation of autobiographical memories was associated with self-reports of negative affect and increased activity in regions known to be involved in self-referential processing, including the sgACC (Kross et al., 2009). Altogether, it could be hypothesized that ruminative thinking interferes with the search or construction of specific memories. In addition, activation of current negative self-schemes might bias memory search towards negative and/or less positive memories (Conway and Pleydell-Pearce, 2000), which in turn might trigger preservative maladaptive thoughts.

Only little is known about the influence of brooding rumination on the occurrence of depression in late-life. Overall, there is evidence that rumination tendency decreases with age (Nolen-Hoeksema and Aldao, 2011; Sütterlin et al., 2012) and that compared to young adults emotionally healthy elderly are better able to disengage from negative information while focusing on positive information (Carstensen, 2006; Reed et al., 2014; Sasse et al., 2014). Intriguingly, such so-called “positivity-effect in aging” is thought to increase resilience against depression in the context of typical age-related challenges (Reed et al., 2014; Mather, 2016). On the other hand, there is also evidence that older people tend to review their past (Ingersoll-Dayton et al., 2010), so one could speculate that if older individuals have an increased brooding tendency they might carry a particularly high risk for becoming depressed when being confronted with age-specific limitations. Indeed, recent data in large samples across the life-span indicate brooding rumination to predict lower life-satisfaction especially in late life (Sütterlin et al., 2012). Other studies in this context have focused on the investigation of regret responsiveness, which is thought to be closely linked to brooding rumination (Savitsky et al., 1997; Stewart and Vandewater, 1999). They could demonstrate a particularly negative influence of regret responsiveness on current mood state even in non-depressed elderly (Lecci et al., 1994; Wrosch et al., 2005). In the same vein, we previously observed neurobehavioral correlates of regret-responsiveness to be more crucial for mood state in old compared to young age (Brassen et al., 2012). In sum, these findings suggest an existing ruminative response style in late life to represent a high-risk factor for depression.

In the present study, we investigated whether brooding tendency in non-depressed older adults is associated with neurobehavioral dysfunctions during autobiographical memory retrieval typically observed in younger depressives. Due to the discussed crucial role of positive memories for well-being in aging, we were particularly interested in brooding effects on memories in response to neutral to positive cues. To this end, we investigated an extensively characterized sample of older, non-depressed participants with varying degrees of brooding tendency using functional magnetic resonance imaging (fMRI) combined with a slightly modified version of an established Autobiographical Memory Cue Word Paradigm (Holland et al., 2011). This paradigm allows for the distinction between: (i) a construction phase, in which participants have to mentally search for specific life episodes based on an initial cue specification; and (ii) an elaboration phase, in which the constructed episode is being re-lived by accessing episodic elements (Conway et al., 2003; Conway, 2009). The expected underlying neural autobiographical memory network comprises primarily left-lateralized medial and lateral prefrontal brain regions which are critical for initiating and monitoring the retrieval process (Svoboda et al., 2006; Cabeza and St Jacques, 2007). The hippocampus (HC) is thought to coordinate specific sensory and perceptual details stored in cortical regions including precuneus, cuneus and other regions of the occipital and parietal lobes (Moscovitch et al., 2006; McCormick et al., 2015). The contributions of emotion have been linked to the amygdala. Self-referential processes, which are crucial in the present study, have been related to sgACC engagement (Cabeza and St Jacques, 2007).

Based on aforementioned findings in younger adults, we expected older participants with brooding tendency to retrieve autobiographical memories with less detailedness and less positive valence. On the neural level, we used factorial and functional coupling analyses to place a special focus on networks related to maladaptive self-referential thinking including the sgACC.

## Materials and Methods

### Subjects

Twenty-two older adults (17 female; mean age 65.1 ± 4.6 years) participated in this study (Table 1). Participants were recruited via newspaper announcements and existing databases. Exclusion criteria were current or previous psychiatric or neurological disorders, severe physical illness at present, current psychopharmacological medication as well as MR-specific exclusion criteria. Medication status during the study was as follows: low doses of antihypertensives (*n* = 4), antihyperglycemic agents (*n* = 2), hormone therapy (*n* = 2). To exclude probable subclinical dementia syndromes or depressive disorders, all older participants underwent a detailed neuropsychological (including Montreal Cognitive Assessment (MoCA) with a cut-off of <26) and psychiatric assessment (including the Mini-International Neuropsychiatric Interview (M.I.N.I.) and Beck Depression Inventory (BDI) with a cut-off of >17). The local ethics committee approved the study, and all participants gave written informed consent and were financially compensated for participation.

**Table 1:** **Demographics**.

	*N* = 23
Age (mean ± SD years)	65.1 ± 4.6
Sex (female/male)	17/5
Montreal cognitive assessment scale (mean ± SD)	27.7 ± 1.4
Beck depression inventory (mean ± SD)	5.0 ± 4.1

### Testing and Questionnaires

#### Brooding

Ruminative thinking in terms of the repetitive “comparison of one’s current situation with some unachieved standard” (Treynor et al., 2003) was assessed using the subscale “Brooding” of the German version of the Response Styles Questionnaire (RSQ-D; Huffziger and Kühner, 2012). The subscale consists of five items, each to be rated by the participants on a scale from 1 (almost never) to 4 (almost always). Participants have to rate the frequency of the following thoughts: (1) “What am I doing to deserve this?”; (2) “Why do I always react this way?”; (3) Thinking about a recent situation, wishing it had gone better; (4) “Why do I have problems other people don’t have?”; and (5) “Why can’t I handle things better?”. Mean brooding score in our sample was 8.27 (SD = 2.8) with a range from 5 to 13. Fifty percent of participants had a score above 7 and thus ranked within the upper third of a large German community sample (score ≥8; Sütterlin et al., 2012).

Emotional stability and current emotional health was assessed with the German short version of the Eysenck Personality Questionnaire (EPQ; Ruch, 1999) and the summarized rating of current life-satisfaction using Questions on Life Satisfaction Modules (FLZM, Henrich and Herschbach, 2000).

#### Autobiographical Memory Cue Word Paradigm

A total of 48 nouns were selected from German word lists (Bascheck et al., 1977; Schwibbe et al., 1981). According to these published norm values (all scales ranging from −20 to +20), selected nouns were highly imaginable (mean 15.31) and concrete (mean 15.03); words were ranging between neutral and positive in valence (mean 4.64). Participants were randomly assigned to one of two parallel versions of 24 stimuli each; words were presented in randomized order. Parallel versions were matched regarding imaginability, concreteness and valence (all *p* > 0.9).

Outside the scanner, participants were instructed that whenever they would be presented with a word together with the instruction “Remember” (maximum 20 s), they should try to recall a specific autobiographical memory that comes into their mind when they think of the cue word. Following established instructions in specific autobiographical memories assessments (Williams et al., 2007), participants were told that a memory is defined as specific if it relates to an event that happened on a particular day and time (no longer than 24 h ) and that occurred at a specific place.

**Figure 1:**
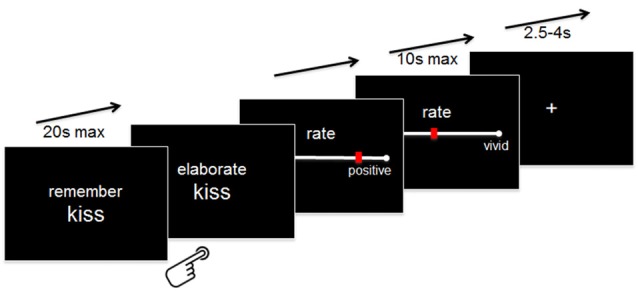
**Experimental paradigm.** Details are provided in the “Materials and Methods” Section.

Specifically, participants were instructed to press a button with their right index finger as soon as they had generated a specific memory. If they were not able to generate a memory within 20 s, a “No memory!”—screen appeared, and the experiment moved to the next word. If they indicated that they had generated a memory, the button press was followed by the instruction “Elaborate” for another 10 s during which they were asked to maintain and elaborate on the details they generated. Following this elaboration phase, they were required to rate the overall valence (“very negative” = 0 to “very positive” = 100) they currently assign to this specific memory and the level of retrieved details (“very vague” = 0 to “very detailed” = 100) by using a continuous VAS pressing buttons with their right index and middle fingers. The rating scales appeared for a maximum of 10 s each. Following the rating scale, a fixation cross appeared for a variable amount of time (range 2.5–4 s; see Figure 1 for an illustration of the paradigm).

All correlation analyses were performed using Spearman’s Rank Correlation using a statistical threshold of *P* < 0.05. In cases of directed hypotheses (i.e., less positive and less detailed memories in brooders, see above), one-sided testing was applied.

### MR Data Acquisition

We used Psychtoolbox software^1^ for stimulus presentation and recording of behavioral responses. MR scanning was performed on a 3-Tesla scanner (Siemens TRIO) at the University Medical Centre Hamburg-Eppendorf, Germany, with a 32-channel head coil. Forty continuous axial slices (2 × 2 × 2 mm, 1 mm gap) were acquired using a gradient echo-planar T_2_*-sensitive sequence (TR = 2590 ms, TE = 30 ms, FOV = 216 × 216 mm^2^, GRAPPA factor 2). After functional imaging, a high-resolution (1 × 1 × 1 mm) T_1_-weighted structural MRI was also acquired for each subject using a three-dimensional magnetization-prepared rapid gradient-echo imaging (3D MP RAGE)sequence.

### Image Analysis

Image preprocessing and statistical analyses were carried out using SPM12^2^. Functional data were corrected for slice-timing, rigid body motion and susceptibility artifacts (“realign and unwarp”). Then, the individual structural T1 image was coregistered to the mean functional image generated during realignment and then segmented into tissue-class images for gray and white matter. Within the DARTEL toolbox, these images were then used to create structural templates as well as individual flow fields which in turn were used for normalization to Montreal Neurological Institute (MNI) space. Data were smoothed with a 6-mm full-width at half maximum (FWHM) isotropic Gaussian kernel.

A two-level random effects approach utilizing the general linear model as implemented in SPM12 was used for statistical analysis. At the single subject level, onsets of construction and elaboration were modeled as separate regressors by convolving delta functions with a canonical hemodynamic response function. Trials in which participants were not able to recall a specific memory (i.e., did not press the button within 20 s after cue presentation) were entered into the model as a separate error regressor. In two additional first level models, construction and elaboration phases were modeled separately, but each including valence and detailedness ratings as parametric modulators.

For each subject, contrast images for each regressor of interest were then entered into a second-level random effects analysis of variance (ANOVA) model. Effects of brooding were investigated by including individual brooding scores as a covariate in second level regression models.

Because sgACC coupling changes have previously been implicated in depressive rumination (Hamilton et al., 2015), we next analyzed functional coupling with the sgACC using a psychophysiological interaction (PPI) analysis. The sgACC-seed region was defined as a 4 mm-sphere centered around the peak *x* = 0, *y* = 26, *z* = −10 obtained from an automated meta-analysis of 95 studies reporting subgenual anterior cingulate activation^3^. Two further first-level models were designed for each participant consisting of the following three regressors: (1) time course of the seed region; (2) the psychophysiological variable (model 1: details ratings, model 2: valence ratings; folded with the hemodynamic response function); and (3) the product of (1) and (2). Again, brooding was entered as a covariate into second level models in order to assess coupling patterns depending on brooding tendency.

As sex was not equally distributed in our sample, gender was included as covariate in all second level analyses. To correct for multiple comparisons, we used anatomical masks of the amygdala and HC from the Harvard-Oxford atlas (probability threshold 50%). The threshold of small volume corrections was set to *P* < 0.05 corrected for multiple comparisons using the family wise error rate (FWE < 0.05). Other regions were reported when passing a whole-brain corrected cluster-threshold of FWE < 0.05 (cluster forming threshold *P* < 0.005 uncorrected).

## Results

### Behavioral Results

As indicated by button presses during the cue word paradigm, participants successfully generated autobiographical memories during scanning for an average of 23.0 out of 24 cue words (SD: 1.3). Across all participants the mean time for constructing a specific memory in response to the cue was 4.8 s (SD: 1.7), mean rating of emotional valence was 67.3 (SD: 8.7) and mean rating of detailedness was 69.6 (SD: 9.4). Intra-individual correlation between valence and detailedness was significant (Fisher’s *z*-score: 0.42, *p* < 0.001), i.e., the more positive the memory was rated, the more details were reported.

Brooding tendency was positively correlated with time for memory construction (*r* = 0.52, *p* = 0.01), i.e., the higher the tendency, the more time was needed to construct a specific memory. Moreover, brooding tendency was negatively associated with ratings of detailedness (*r* = −0.49, *p* = 0.01, one-sided) as well as the emotional valence ratings of elaborated memories (*r* = −0.38, *p* = 0.04, one-sided), i.e., participants with higher brooding tendency rated their memories as less detailed and as less positive.

Emotional stability as measured with the EPQ as well as higher life satisfaction were both correlated with less brooding tendency (*r* = 0.64 and *r* = 0.68, both *p* < 0.001) but were not systematically associated with task-specific parameters.

### Imaging Results

#### Activation Patterns During Memory Construction and Elaboration (Table 2)

**Figure 2:**
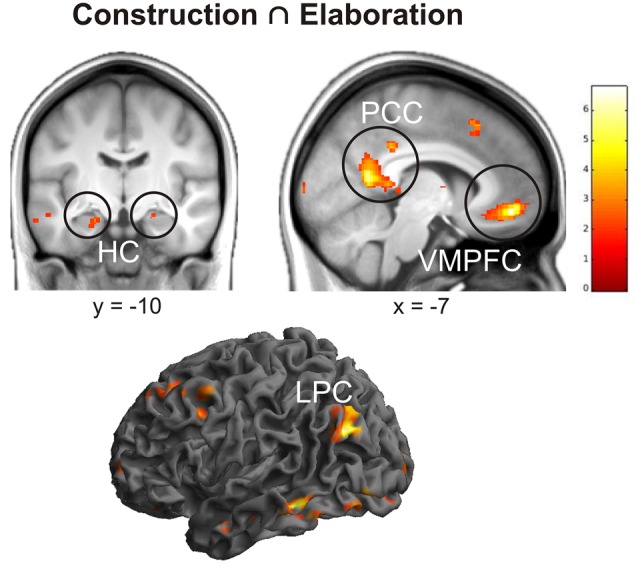
**Results of the conjunction analysis including brain activation during the construction and elaboration phase.** Activations are overlaid on the mean structural image of all participants (visualization threshold *P* < 0.005 uncorrected). All *p* < 0.05 FWE corrected. HC, Hippocampus; PCC, Posterior Cingulate Cortex; VMPFC, Ventromedial Prefrontal Cortex; LPC, Lateral Parietal Cortex.

First of all, we conducted a conjunction analysis to determine which regions were commonly activated during the construction and elaboration phases. Results revealed several left-lateralized regions of the autobiographical network (Svoboda et al., 2006) including posterior cingulate cortex (PCC), lateral parietal cortex (LPC), ventromedial prefrontal cortex (VMPFC), orbitofrontal cortex (OFC) and the bilateral HC (all *p* < 0.05 FWE; Figure 2).

**Table 2:** **Brain regions involved in memory construction, elaboration and modulation by ratings**.

		MNI (peak)		
Brain region	Side	*x*	*y*	*z*	Cluster size	*Z*-Score
**Construction ∩ Elaboration**
Ventromedial prefrontal cortex	L	−6	46	−12	533	4.78
Medial orbitofrontal cortex	L	−38	40	−12	Same Cluster	3.86
Posterior cingulate cortex	L	−8	−56	14	657	4.85
Lateral parietal cortex	L	−38	−76	32	306	4.64
Hippocampus	L	−20	−14	−20	45	3.36
	R	20	−12	−18	13	3.42
**Construction > Elaboration (masked by Construction ∩ Elaboration)**
Superior frontal gyrus	R	24	−4	54	1221	4.58
Cuneus	R	10	−84	10	10991	4.84
Middle occipital gyrus	R	34	−82	8	Same Cluster	4.77
**Construction × Details**
Positive modulation
Hippocampus	L	−32	−18	−16	41	3.51
**Construction × Valence**
Positive modulation
Hippocampus	L	−26	−22	−18	27	3.47
	R	24	−16	−14	28	3.43

In order to assess whether some of these regions are more activated (i.e., quantitative differences) during either construction or elaboration, we then conducted two triple conjunction analyses including in addition to either the contrast construction > elaboration or elaboration > construction. Results revealed none of the aforementioned regions to be significantly more activated in the single phases. In the next step, we investigated whether there are additional (i.e., qualitative differences) regions being differentially activated during construction and elaboration. We therefore exclusively masked (masking threshold: 0.05) the differential contrasts (construction > elaboration and elaboration > construction, respectively) with results from the first conjunction analysis. Here, we found some additional regions to be more activated during construction compared to elaboration including right cuneus, right middle occipital gyrus and the right superior frontal gyrus (SFG). No additional regions were found to be more activated during elaboration compared with construction.

**Figure 3:**
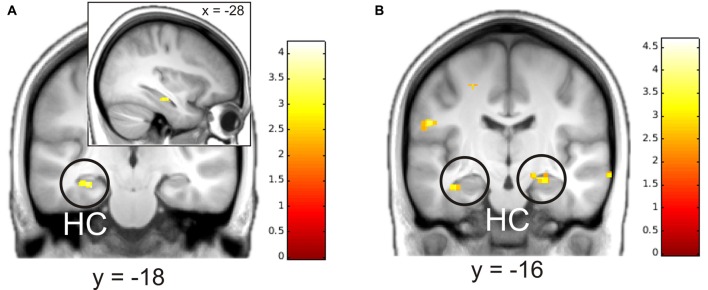
**Parametric modulation of brain activity by ratings of details (A) and valence (B) after the elaboration of memories.** Activations are overlaid on the mean structural image of all participants (visualization threshold *P* < 0.005 uncorrected). All *p* < 0.05 FWE corrected. HC, Hippocampus.

We then analyzed parametric modulation of brain activation by ratings of detailedness and valence. The analysis of the construction phase revealed a significant positive linear modulation by detailedness of left hippocampal activity, i.e., left hippocampal activity predicted detail richness of single memories across all participants (Figure 3). Parametric modulation by valence ratings resulted in bilateral HC activity, i.e., the higher the hippocampal signal during construction the more positive was the emotional rating. No significant modulation results emerged for the elaboration phase.

#### Interactions With Brooding (Table 3)

**Figure 4:**
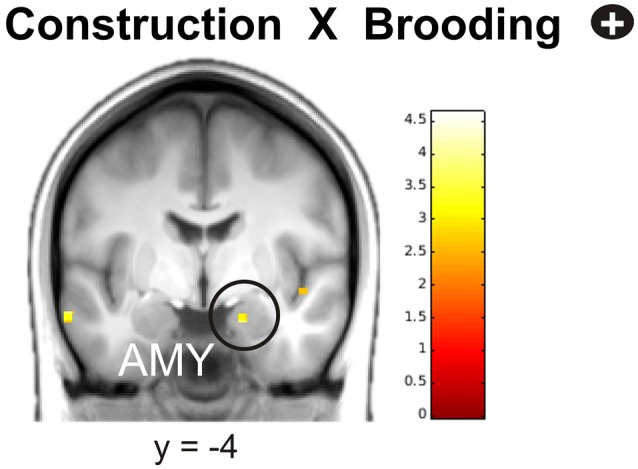
**Positive correlation of brooding tendency with right amygdala activation during memory construction.** Activations are overlaid on the mean structural image of all participants (display threshold *P* < 0.005 uncorrected). *P* < 0.05 FWE corrected.

In the next step, we investigated the relationships between neural activity during memory construction/elaboration and brooding tendency. Results revealed a positive correlation between brooding tendency and activity in the right amygdala during the construction phase (Figure 4).

**Table 3:** **Brain regions which show interactions with brooding tendency**.

		MNI (peak)		
Brain region	Side	*x*	*y*	*z*	Cluster size	*Z*-Score
**Construction × Brooding**
*Positive correlation*
Amygdala	R	18	−4	−22	15	3.08
**Elaboration × Brooding**
*Negative correlation*
Superior frontal gyrus	R	10	58	34	419	3.58
Medial frontal gyrus	R	18	32	34	Same cluster	3.48
Inferior frontal gyrus	L	−44	8	30	1902	3.91
Superior temporal gyrus	L	−62	−6	8	Same cluster	4.52
	R	54	−26	16	301	4.36
Precuneus	L	−14	−70	24	365	3.62
**sgACC Coupling × Details × Brooding**
*Positive correlation*
Hippocampus	L	−26	−22	−12	15	3.52
**sgACC Coupling × Valence × Brooding**
*Negative correlation*
Amygdala	R	18	−4	−20	10	3.21

**Figure 5:**
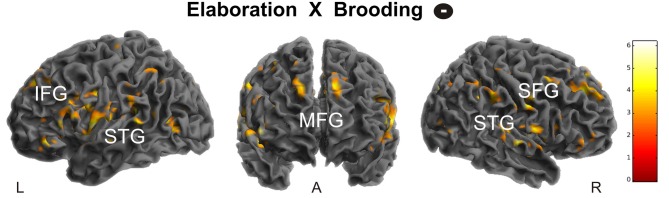
**Negative correlation of brooding tendency with fronto-temporal activation during memory elaboration.** Activations are overlaid on the mean structural image of all participants (display threshold *P* < 0.005 uncorrected). All *p* < 0.05 FWE corrected. IFG, Inferior Frontal Gyrus; STG, Superior Temporal Gyrus; MFG, Medial Frontal Gyrus; SFG, Superior Frontal Gyrus.

During elaboration, brooding tendency was significantly negatively correlated with activation in a cortical network including the bilateral superior temporal gyrus (STG), precuneus, right superior and medial frontal gyrus (MFG) and the left inferior frontal gyrus (IFG; Figure 5).

**Figure 6:**
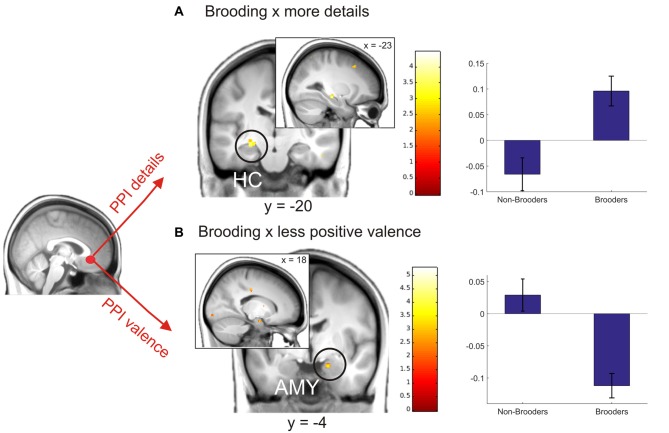
**Coupling modulation by details (A) and valence (B) depending on brooding tendency.** The right plots depict parameter estimates of this analysis for non-brooders (*N* = 11) and brooders (*N* = 11) separately, using median split (≥8) on brooding scores. Activations are overlaid on the mean structural image of all participants (display threshold *P* < 0.005 uncorrected). All *p* < 0.05 FWE corrected.

Based on recent evidences for a changed connectivity pattern of the sgACC in younger depressed ruminators, in the next step we extracted the deconvolved fMRI-time-course from a predefined sgACC seed region (for details see “Materials and Methods” Section) and used PPI including: (a) detail ratings; and (b) valence ratings. Since we expected changed coupling patterns depending on brooding, we then entered brooding tendency as a regressor in the second level analyses of PPI-interaction contrasts. Results revealed a positive correlation between brooding tendency and increased coupling by detailedness of the sgACC with the left HC. As indicated by the separate consideration of parameter estimates for brooders and non-brooders using median split (brooding tendency ≥8), this result was driven by both detailed memory retrieval in brooders, were related to an increased sgACC-HC coupling and the inverse pattern emerged for non-brooders (Figure 6A).

Coupling analysis including valence modulation revealed a relation between brooding tendency and coupling with the right amygdala. Visual inspection of median split results indicates this result to be mainly driven by an increased coupling in brooders when memories were retrieved as less positive (Figure 6B).

## Discussion

In our study, healthy older brooders demonstrated a neurobehavioral response pattern during autobiographical memory retrieval that strikingly overlaps with previously reported dysfunctions observed in young depressive adults (e.g., Dalgleish and Werner-Seidler, 2014; Young et al., 2016). Specifically, brooders needed more time to construct an autobiographical memory event and rated their memories as less detailed, possibly due to interference by ruminative thinking. Moreover, older participants with high brooding tendency perceived their retrieved memories as less positive, which might be a consequence of negatively biased self-schemes. This is supported by our neural findings of: (i) stronger engagement and coupling of a network consisting of key regions related to maladaptive self-focused thinking; and (ii) reduced activation in cortical autobiographical memory circuits.

To our knowledge, this is the first study investigating the neural relation of brooding with both autobiographical memory construction and elaboration. Our paradigm resulted in a strong activation of the autobiographical memory network, highlighting the validity of our task. During the construction phase, we found left hippocampal activity to be related to the reported detailedness and bilateral HC activity to code the valence of the memories. This is in line with previous research highlighting the role of the HC in paving the way to perceptual details of autobiographical memories (Nadel and Moscovitch, 1997; Addis et al., 2004) and in emotional processing (Robinson et al., 2015).

Participants with higher brooding tendency reported their memories as less positive and demonstrated an increased activity in the right amygdala during memory construction compared to participants with lower brooding tendency. Interestingly, recent findings indicate altered amygdala activity during autobiographical memory recall to represent both state and trait markers of depression in younger age (Young et al., 2016). More specifically, our results also fit with research indicating that brooding and associated negative mood is related to a difficulty to find specific positive memories (Williams et al., 2007). In addition, sgACC-amygdala coupling directly predicted less positive memories in brooders. It has been speculated that maladaptive self-referential processes underlying rumination hinder adequate memory retrieval and embed memories in a more negative context (Sperduti et al., 2013; Dalgleish and Werner-Seidler, 2014). Support for this assumption comes from findings of increased sgACC activation during autobiographical memory elaboration after rumination induction in healthy young adults (Kross et al., 2009). In a similar vein, sgACC-amygdala coupling during resting state fMRI in adolescence was found to be related to negative affectivity (Davey et al., 2015) and was elevated in younger depressives compared to controls (Connolly et al., 2013). As far as we know, the current study is the first that extended these findings to participants with an existing brooding tendency and to the active construction of autobiographical memories. Since we used neutral to positive cues in our paradigm, such processes rather ended in less positive than more negative memories. Given large evidence that a focus on positive memories is critical for emotional well-being in late-life (Brassen et al., 2011; Mather, 2016) and findings that younger individuals at risk for depression demonstrate neural changes during positive autobiographical memory retrieval (Young et al., 2016), reduced positivity in older brooders might put them at particularly high risk for depression.

Consistent with findings in younger depressed ruminators, brooding in healthy older adult was related to impaired autobiographical memory retrieval in terms of longer construction times and lower detail ratings (Schoofs et al., 2013; Hamlat et al., 2015). While longer construction phases might result from interference by negative, self-referential thinking, our finding of decreased activation patterns in autobiographical memory networks during elaboration might mirror reduced perception of detail richness. Especially the IFG plays an important role in appropriate information selection (Badre and Wagner, 2007), emotion regulation (Ochsner et al., 2012) and controlled retrieval, for instance by diminishing the impact of negative goal-irrelevant emotional distraction (Dolcos et al., 2013) or the voluntary up-regulation of positive emotions (Kim and Hamann, 2007). The fact that brooders showed a decreased activation of this region during memory elaboration might point to a less controlled, maybe inappropriate selection of memory details resulting rather from rigid self-schemes than appropriate and cohesive selection. The IFG has been shown to be sensitive to age-related changes especially during the elaboration of autobiographical memories (St Jacques et al., 2012). Thus, it could be speculated that such decline triggers potentially negative effects of brooding tendency in aging.

While sgACC-HC coupling was negatively related to detail ratings in non-brooders, the opposite pattern emerged for participants with high brooding tendency. Due to the small sample sizes of subgroups we can only speculate on this finding. One interpretation could be that non-brooders engaged cognitive control to suppress an impact of potentially interfering self-referential processes on detailed memory retrieval. Detailed memories in brooders, in contrast, might activate (negative) self-schemes which in turn might further promote persistency and re-encoding of distorted memories. Indeed, the sgACC and the medial temporal lobe (MTL) are strongly, reciprocally connected and their coupling is enhanced in younger patients with major depressive disorder (de Kwaasteniet et al., 2013). The testing of such causal connectivity hypotheses, however, requires larger subsamples. In any case, the association between the sgACC and regions of the MTL seems to be crucial for mood state and rumination. This might become especially relevant in the context of age-related changes in fronto-limbic network integrity (Brassen et al., 2008; Lehmbeck et al., 2008; Fjell et al., 2009). For example, the “vascular depression” hypothesis posits that white matter lesions, caused by ischemic changes, are the main cause for the frequent comorbidity between vascular disease and depression in aging (Taylor et al., 2013). Lesions are primarily located in target fronto-subcortico circuits such as the cingulum bundle (Taylor et al., 2014). Hence, one could speculate that disruptions in critical neural emotion-regulation circuits due to vascular disease hinder successful adaptation in aging. In our sample, four participants with varying degree of brooding tendency (individual scores: 7, 8, 12, 13) took low doses of antihypertensive medication which rather argues against a significant influence of vascular disease on our findings. However, since we did not use specific MRI sequences sensitive for hyperintensities (e.g., Fluid-attenuated inversion recovery [FLAIR] sequence), an impact of subtle micro-structural changes on our findings (e.g., Popa-Wagner et al., 2014) cannot be excluded.

It should be noted that this first study specifically focused on depression-like neurobehavioral patterns in older adults and not on age-effects in general, e.g., regarding autobiographical memory functions (St Jacques et al., 2012). The overlap of our findings with reports on neurobehavioral changes in younger ruminators is striking but, of course, exact common or specific patterns can only be investigated by including a young age group.

Moreover, in line with previous studies on the emotional evaluation of autobiographical memories (e.g., Greenberg et al., 2005; Bado et al., 2014) we only assessed the overall value participants assigned to their retrieved memory using well-established bipolar dimension scales (e.g., Bradley and Lang, 1994). In order to get a more complete picture of emotional and mood aspects, participants relate to previous events; in future studies scanning sessions could be followed by detailed autobiographical memory interviews in which memories have to be evaluated on different subscales related to both positive and negative affect (e.g., The Positive and Negative Affect Schedule, PANAS; Watson et al., 1988).

Altogether, our findings underline brooding tendency in aging as a critical indicator for maladaptive self-referential processes when it comes to autobiographical memory retrieval. Aging has been related to an increased tendency to review the past (Ingersoll-Dayton et al., 2010) and disengagement from unreached goals as well as a focus on the positive is crucial for well-being in late life (Heckhausen et al., 2010; Mather, 2016). Thus, ruminating upon previous decisions and counterfactual thinking might be particularly maladaptive in older age, especially in the context of age-related changes in fronto-limbic brain circuits and executive control. Recent data demonstrate beneficial intervention effects on autobiographical memory function and rumination in younger age (Raes et al., 2009, 2012; van Vugt et al., 2012; Bratman et al., 2015), e.g., by applying a memory specificity training (MEST; Raes et al., 2009) or nature experiences (Bratman et al., 2015). Targeting older peoples’ autobiographical memory ability in prevention and intervention studies might therefore be a promising approach to increase resilience against late-life depression.

## Author Contributions

SB developed the study design. SB and SS jointly designed the experiment. SS programmed the paradigm and she collected and preprocessed all data. SB conducted the final behavioral and neuroimaging analyses and prepared the manuscript.

## Conflict of Interest Statement

The authors declare that the research was conducted in the absence of any commercial or financial relationships that could be construed as a potential conflict of interest.

## Footnotes

^1^http://psychtoolbox.org/

^2^http://www.fil.ion.ucl.ac.uk/spm/

^3^http://neurosynth.org
